# Solar drying of granulated waste blends for dry biofuel production

**DOI:** 10.1007/s11356-021-12848-3

**Published:** 2021-02-26

**Authors:** Małgorzata Wzorek

**Affiliations:** Department of Process and Environmental Engineering, Faculty of Mechanical Engineering, Opole University of Technology, ul. Mikołajczyka 5, 45-271 Opole, Poland

**Keywords:** Solar energy, Drying, Greenhouse, Sewage sludge, Biofuel, Waste

## Abstract

In the paper, results of drying biofuels from sewage sludge using solar energy are presented. Drying rates of biofuels made from sewage sludge and coal slime (PBS), sewage sludge and meat and bone meal (PBM), and sewage sludge and sawdust (PBT) with 15-mm and 35-mm granule particle size were studied. Tests were performed in a solar greenhouse dryer equipped with a specially designed mixing system. Experiments were aimed at determining the drying time of biofuels under various weather conditions in the southwestern part of Poland. In summer, in order to determine the best conditions for drying, tests were performed using various parameters, i.e., layers of various thickness, such as 5, 10, and 20 cm, and various mixing intensity (no mixing, mixing 3 and 5 times/day). In spring and the fall, 10-cm thick layers combined with 5 times mixing of fuels per day were used. The performed tests demonstrated that it is beneficial to dry fuels in 10-cm thick layer. In spring and the fall, PBS and PBM biofuels laid out in layers with just such thickness showed moisture content reduced to less than 10% after 8 days, while the PBT biofuel reached the same level after 14 days. In summer, the same result may be obtained for all the biofuels after 4 days on average. The presented original method of solar drying of biofuels obtained from sewage sludge and other waste may be used in wastewater treatment plants which process sewage sludge into fuels without incurring any additional costs for supplying heat.

## Introduction

Sewage sludge management is one of the significant challenges of wastewater management in Poland. Sewage sludge represents a major type of waste produced by wastewater treatment plants and accounts for roughly 1–2% of the total amount volume of wastewater treated. Its disposal can add significant energy demand to the already high requirements of a treatment plant (Capodaglio and Olsson 2020). An amount of sewage sludge cannot be prevented and is disposed in line with the requirements regarding the quality of treated sewage.

A total of 3253 municipal wastewater treatment plants were operating in Poland in 2019, which is almost three times as many when compared to 1995, serving more than 28.5 million residents (i.e., 73.5% of the total population (Statistics Poland 2018).

Wastewater treatment plants use ever more efficient treatment systems, such as biological treatment with extended nutrients removal (three-phase and double-phase, hybrid, cyclical, semi-cyclical, or single-phase systems with chemical phosphorus precipitation). The resulting relationship is quite simple—the more sophisticated waste treatment technologies, the more sewage sludge is generated which is additionally harder to dispose of.

According to data published by Statistics Poland (2018), 613,000 t of dry mass of sewage sludge were produced in Poland by municipal wastewater treatment plants in 2016, additionally around 11,000 t were generated by domestic sewage treatment systems in rural areas (Pawlita-Posmyk and Wzorek 2016).

The amount of generated sewage sludge depends on many factors, mainly on the content of organic pollutants in the sludge and—as mentioned earlier—on the technology of its treatment.

The varying composition and amounts of micropollutants in the sewage sludge, such as polycyclic aromatic hydrocarbons (PAHs), heavy metals, polychlorinated biphenyls (PCBs), and pathogens make the sludge management problems difficult to solve (Ozaki et al. 2017; Mailler et al. 2014; Raheem et al. 2018; Pawlita-Posmyk and Wzorek 2017). A major contributing parameter here is attributable to the high degree of sludge hydration which in the case of raw sludge amounts to more than 99%, with between 80 and 65% for mechanically dehydrated sludge.

One of the methods applied to significantly reduce both the mass and volume of sewage sludge involves thermal processes, such as combustion, pyrolysis, or gasification (Fonts et al. 2012; Oladejo et al. 2019). Pyrolysis and gasification, in addition to mass reduction, can also generate liquid or gaseous fuels for subsequent use (Capodaglio et al. 2016); these can be of particular economic interest in view of a recent EU transportation policy (Raboni et al. 2015).

The current role and immediate prospects of biofuels production from waste products including sewage sludge, wood and coal residues, and other organic waste are the topic of intensive research today (Callegari et al. 2020).

However, the fundamental applicability of sewage sludge in the energy-rich biofuels generation process is associated with the necessity of fulfillment of requirement of low moisture content.

Sewage sludge containing 20–30% of dry mass may only be incinerated after another fuel has been added, while autothermal combustion will occur only after the mass has been partially dried up to 50% (Werther and Ogada 1999). Drying the sewage sludge up to roughly 10% of dry mass makes it suitable for application in coal co-combustion processes, such as power and heat plants and waste incineration plants or in the process of cement kiln production (Murakami et al. 2009; Wasielewski et al. 2013; Wzorek and Troniewski 2007).

Sewage sludge constitutes the type of waste for which the boundary value of mechanical dehydration is determined with the content of wastewater solids in the range of 30 ÷ 35%. The higher degree of dehydration, equal to 90%, may only be achieved through thermal drying. This implies that a substantial amount of water must be evaporated, which is energetically expensive and could offset the advantages of energetic recovery from the combustion.

## Drying of biosolids: thermal vs. solar

Thermal drying is frequently used in the processing of biomass and waste into fuels: lowering the moisture content improves their energy characteristics—improvement of the low heating value and acquiring physical properties that results in lower transport and warehousing costs. In the case of organic waste, drying is also used to improve hygienization of the product; however, the process contributes to higher production costs.

Owing to the ever more popular application of waste, biomass, and sewage sludge, an increased interest in the study of drying has been observed, with high-temperature drying processes (Collard et al. 2017; Perazzini et al. 2016), biological drying (Ab Jalil et al. 2016; Zhang et al. 2018), and the potential for using alternative sources of energy to this end being considered (Singh et al. 2018; Maurer and Müller 2019).

The conventional processes of drying require a considerable amount of energy. For example, in the case of sewage sludge, and depending on the drying method, the water evaporation may require to either 0.7–0.9 kWh/kg or even with the expected energy consumption ratio to be of up to 1.4 kWh/kg (Bennamoun et al. 2013; Tańczuk and Kostowski 2021).

An alternative to the thermal drying methods is drying with the application of solar energy. Literature on the subject provides extensive information about the research into the application of solar energy to dry agricultural crops and wood both in solar-drying plants and outdoors (VijayaVenkataRaman et al. 2012; Campean and Marinescu (2011) and recently, also to dry waste, biomass, and sewage sludge (Tun and Juchelková 2019; Alamia et al. 2015; Mehrdadi et al. 2007; Bennamoun 2012).

In Europe, a number of solar sewage sludge dryers have been constructed, mainly in Germany, France, Austria, and Switzerland, but also in Poland (I IST-Anlagenbau GmbH 2020; Thermo-system. Industrie-&Trocknungstechnik GmbH 2020; Bożym and Bok 2015).

Drying technologies based on solar energy in solar dryers utilize the greenhouse effect produced inside a structure covered with a sunlight-transmitting material.

Solar dryers used to dry sewage sludge feature a greenhouse structure covered with glass, foil, or polycarbonate panels (Singh et al. 2018).

The drying rate depends on a number of factors, including weather, such as the temperature and relative humidity of the air, solar radiation intensity, the heating medium flow direction and speed, the size of the uncovered area of the material to be dried, and the bed thickness (Luboschik 1999; Yoo et al. 2017; Hii et al. 2012).

One of the key parameters of the drying process consists in the relative humidity of the drying air. The lower the relative humidity of the drying air, the more water evaporates from the product, ensuring a lower moisture content of the final product (Helwa et al. 2004). Shin et al. (2000) indicate that greater influence of drying rate is presented by relative humidity instead of its temperature. Luboschik (1999) has estimated that as long as the partial pressure of water vapor in the air depends only on the absolute humidity rather than the air temperature, the best results are obtained by drying with the warm sludge and dry air. For those reasons, solar dryers feature exchange air systems, such as air supply and air exhaust.

Undoubtedly, solar irradiance represents a most important factor in the solar drying since it is the basic source of solar energy required to dry the material at hand.

The prevailing weather conditions in Poland favor utilizing the solar energy available. The strategy for using renewable energy resources even assumes the technical potential for using solar radiation energy may reach 1340 PJ (**Polish’s energy policy until 2030 **2009**)**.

**Fig. 1:**
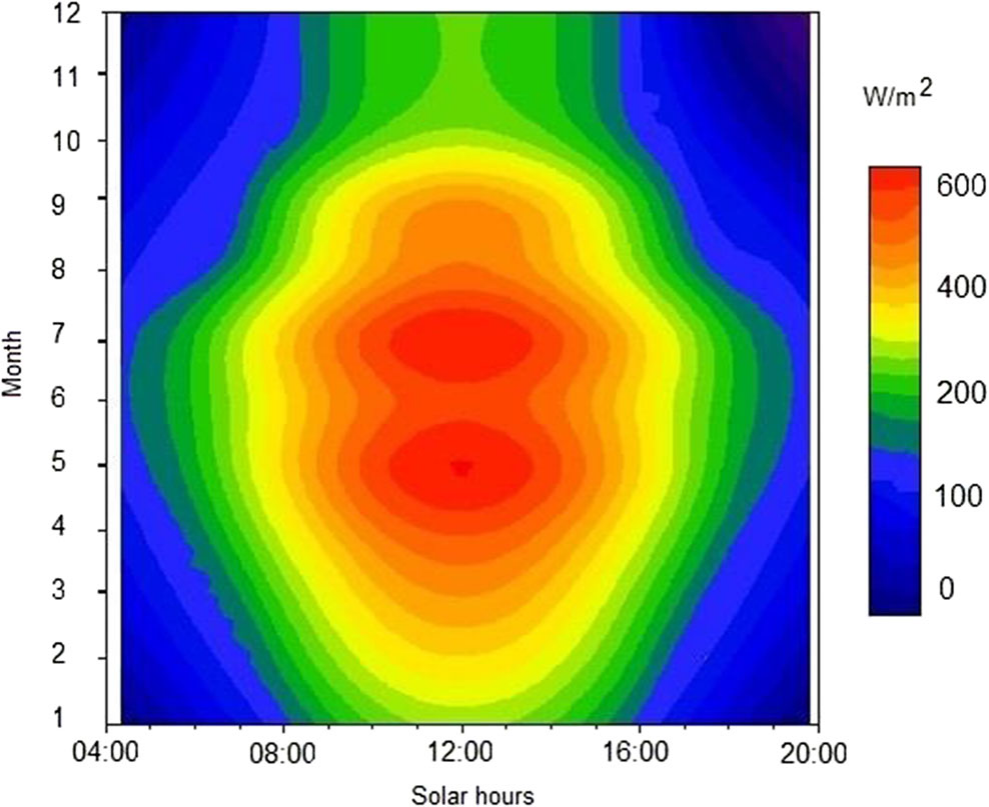
A 24-hour average distribution of solar radiation intensity for Opole (southwestern part of Poland)

**At this latitude, the average annual irradiation time equals roughly 1600 h while the annual solar radiation density ranges from 956 to 1100 kWh/m**^**2**^ **(EU Science Hub** 2020**)**. The 24-h average distribution of solar radiation intensity for Opole (southwestern part of Poland) is presented in Fig. 1.

Another factor that increases contact between the material to be dried and the drying medium is the mixing system. Various types of mixing equipment are used to dry the sewage sludge only, such as chain or shovel turners, electric moles, or a prism system for turning the sludge.

The systems also call for a different thickness of the material to be dried which—in the case of Thermo-system®—varies from 1 to 10 cm (Thermo-system); for the Wende Wolf®, it is between 30 and 40 cm (IST-Anlageenbau GmbH); and in the case of prism technologies, it may even be as high as 50 cm (Trojanowska 2016).

In Europe, solar dryers mostly start operating in spring, continue in summer, and close down in the fall. Only those plants which have been additionally equipped with an underfloor heating installation can operate in winter. The evaporating efficiency factor of the solar dryers ranges from 590 to 792 kg_H2O/_m^2 .^ a under the conditions prevailing in Poland; while in the case of hybrid dryers (with an additional source of heat), it stands at 856–927 kg_H2O_/m^2 .^ a (Trojanowska 2016).

The technologies currently available in the market which mix the sludge during the drying in solar dryers are used to dry the sewage sludge only, a process that delivers dry granulate of up to 5 mm in size and 10–20% moisture content.

Depending on its calorific value (>12 MJ/kg), dried sludge may be used for combustion processes, among others. Nevertheless, sewage sludge in which energy characteristics is too low may be used together with other types of waste and include other fuel granulates with appropriate energy and physical parameters.

No information has been found either in the available literature or in the market about solar greenhouse drying of granulated biofuels. Attempts have been made to solar dry coffee pulp (Cubero et al. 2014) for pellet production in open area conditions or olive waste (Maragkaki et al. 2016), wood chips (Perea-Moreno et al. 2016), and RDF fuel (Trojanowska 2016) in the solar greenhouse dryers. However, the wastes were dried in a shredded form. To this end, attempts have been made to develop a new technology of drying granulated biofuels made from sewage sludge, a process requiring designing a new mixing system and defining proper conditions for handling the drying process.

The paper presents research on the determination of optimal solar-drying conditions for granulated biofuels from sewage sludge using the proprietary technology of mixing in a solar dryer.

## Materials and methods

### Materials

Feedstocks obtained from municipal sewage sludge and other waste, such as coal slime, meat and bone meal, and sawdust, have been solar-dried. The method of biofuels production depends on mixing the components in specific proportions then forming granulate in the rotary drum. The drum is equipped with a feeder system ensuring the granulate diameter in the range from 15 to 35 mm (Wzorek 2008).

The following biofuel compositions were tested:

- 60 wt.% of sewage sludge, 34 wt.% coal slime, and 6 wt.% of quicklime - PBS,
- 75 wt.% of sewage sludge, 24 wt.% of meat and bone meal, 1 wt.% of quicklime - PBM,
- 80 wt.% of sewage sludge, 19 wt.% of sawdust and 1wt.% of quicklime - PBT.

Table 1 shows the biofuel parameters. Following the mixing and granulation operations, biofuels contain between 43 and 68% of moisture.

**Table 1:** Properties of biofuels from sewage sludge

Parameter	Unit	PBS	PBM	PBT
Higher heating value, HHV	MJ/kg	21.71	15.97	15.54
The initial moisture content	%	43–49	49–54	60–68
Voltaire matter^1^	% d.m.	34.44	55.29	59.87
Ash^1^	% d.m.	27.26	33.72	20.36

*d.m.* dry mass

^1^Source: Wzorek 2020

In order to define how the granulate size affects the drying rate, biofuels with 15-mm and 35-mm granulations were tested.

### Methods

**Fig. 2:**
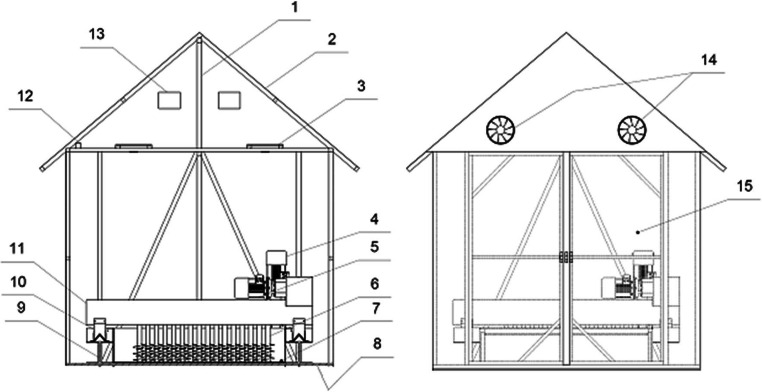
Solar greenhouse dryer scheme: 1 drying plant structure, 2 polycarbonate coating, 3 forced draft fans, 4 traveling beam drive, 5 mixer paddle drive, 6 rotating vertical paddles, 7 drying area, 8 concrete floor, 9 external structure of the mixing device, 10 tracks, 11 beam, 12 air temperature and humidity sensor, 13 exhaust flaps, 14 exhaust fans, and 15 entrance to greenhouse dryer

Biofuels were dried in the solar dryer with a total area of 15 m^2^, as shown in Fig. 2.

The solar dryer was located in Opole, southwestern part of Poland (37° 51′ N latitude, 27° 51′ 66 E longitude) in the Opole University of Technology campus.

The drying plant structure (1) was coated with 5-mm thick multi-chamber polycarbonate (2). The plant features two automatic sensors recording both the air temperature and humidity. The solar dryer was also equipped with an air exhaust system comprising six forced draft fans (3) with 624 m^3^/h capacity and two exhaust fans (14) with 95 m^3^/h capacity. The forced draft fans were mounted in two rows in the upper part of the drying plant, while the exhaust fans were installed on the front wall. The forced draft fans are designed to circulate the air inside the dryer in order to improve mass and heat exchange, whereas the exhaust fans are designed to expel humid air from inside the dryer. The air humidity inside the greenhouse was monitored using a hygrometer. Once the designed relative humidity level (75%) was exceeded, the ventilation flaps would open and the draft fans start working.

A mixing device was installed in the solar dryer (both the device itself and the manner of mixing are protected under a patent (Wzorek and Głowacki 2014). The device consists of a moving beam (11) with 15 vertical paddle mixers mounted thereon (6); they are shaped so as to lift the material being dried. The mixers are arranged in two rows so as to cover the entire working area. Both the rotation speed of the vertical mixers and the travel speed of the traveling beam are adjustable with a frequency converter. Research into solar drying of biofuels produced from sewage sludge was conducted under various weather conditions, in spring, summer, and the fall. Based on the experience of the existing sewage sludge solar greenhouse dryers, which do not operate in winter time, no drying was performed during that time.

During summer, a period most suitable for determining the best parameters for solar drying, testing was carried out on biofuel beds of varying thickness: 5, 10, and 20 cm, alternating the mixing speed, namely option I no mixing, option II mixing 3 times/day, and option III mixing 5 times/day.

Testing of 35-mm granulated biofuel carried out in the fall and spring involved defining fixed operation of the mixing device with the beam traveling along the entire dryer length 3 times per day of drying, and the vertical mixer rotational speed of 15 rpm.

Biofuel samples were taken every day at 6 PM to determine fuel moisture reduction. Moisture levels were measured as stipulated in the PN-80 G-04511 standard. The mixer was not activated on the first day of testing and mixing started only once the fuel top layer had dried up.

Biofuel moisture variations over time were expressed approximately with the following regression function:

$$ f\left(X,P\right)=W(t)={ae}^{- bt} $$

where *W*(*t*) is the moisture variation as a function of time, %; *a*_*i*_, *b*_*i*_, and *b*_*i*_ estimates of the regression function structural parameters; and *t* time, day.

## Results and discussion

**Fig. 3:**
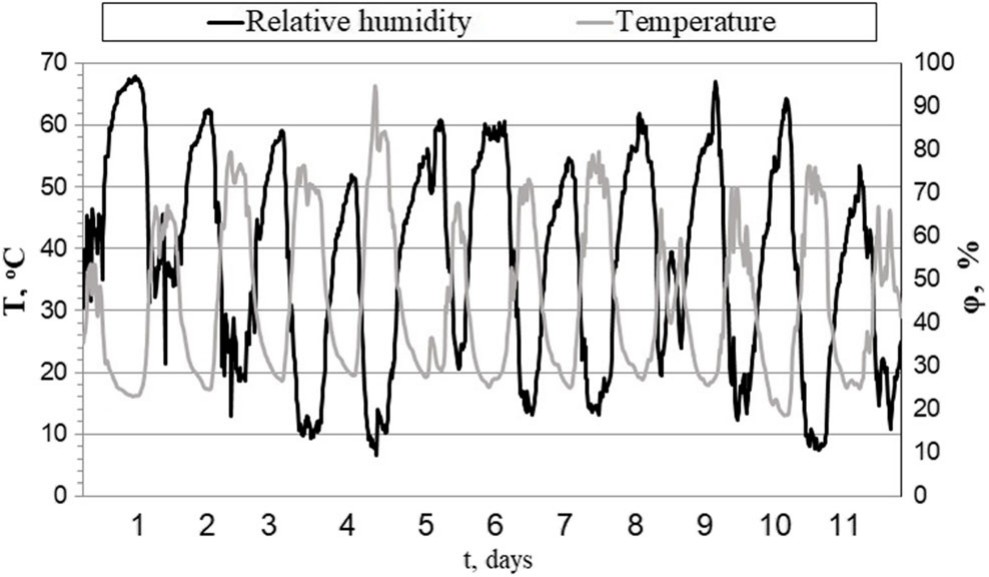
Relative temperature and humidity of air in the solar dryer in summer test

**Fig. 4:**
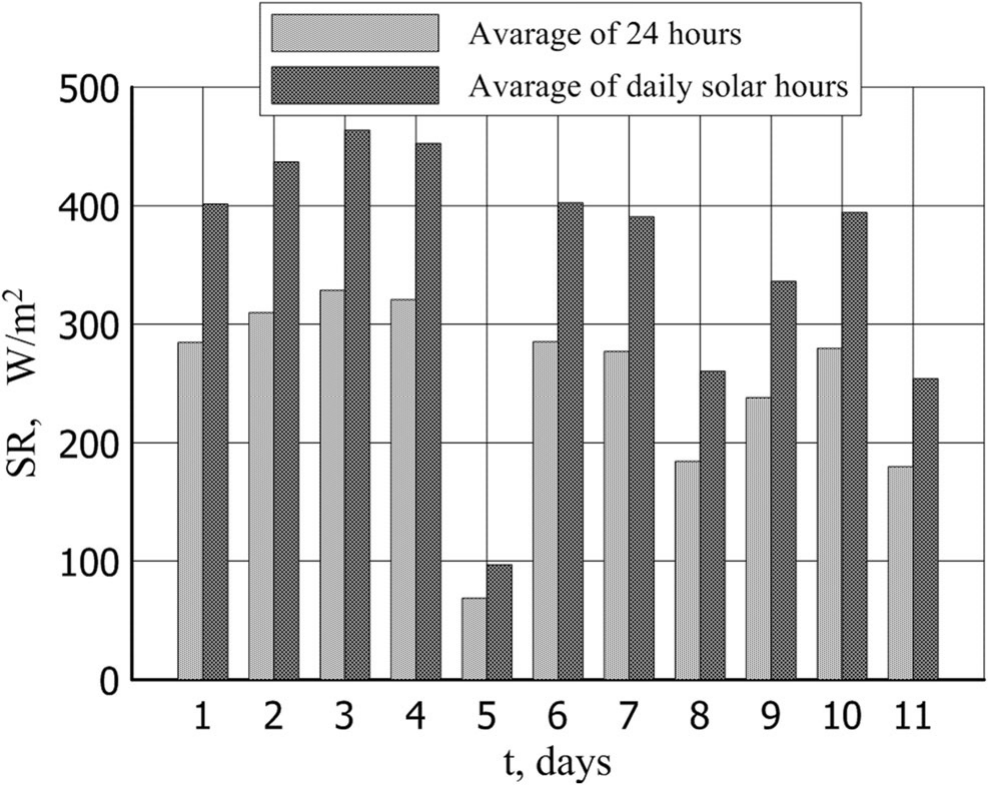
Total solar radiation (SR) in summer test

**Fig. 5:**
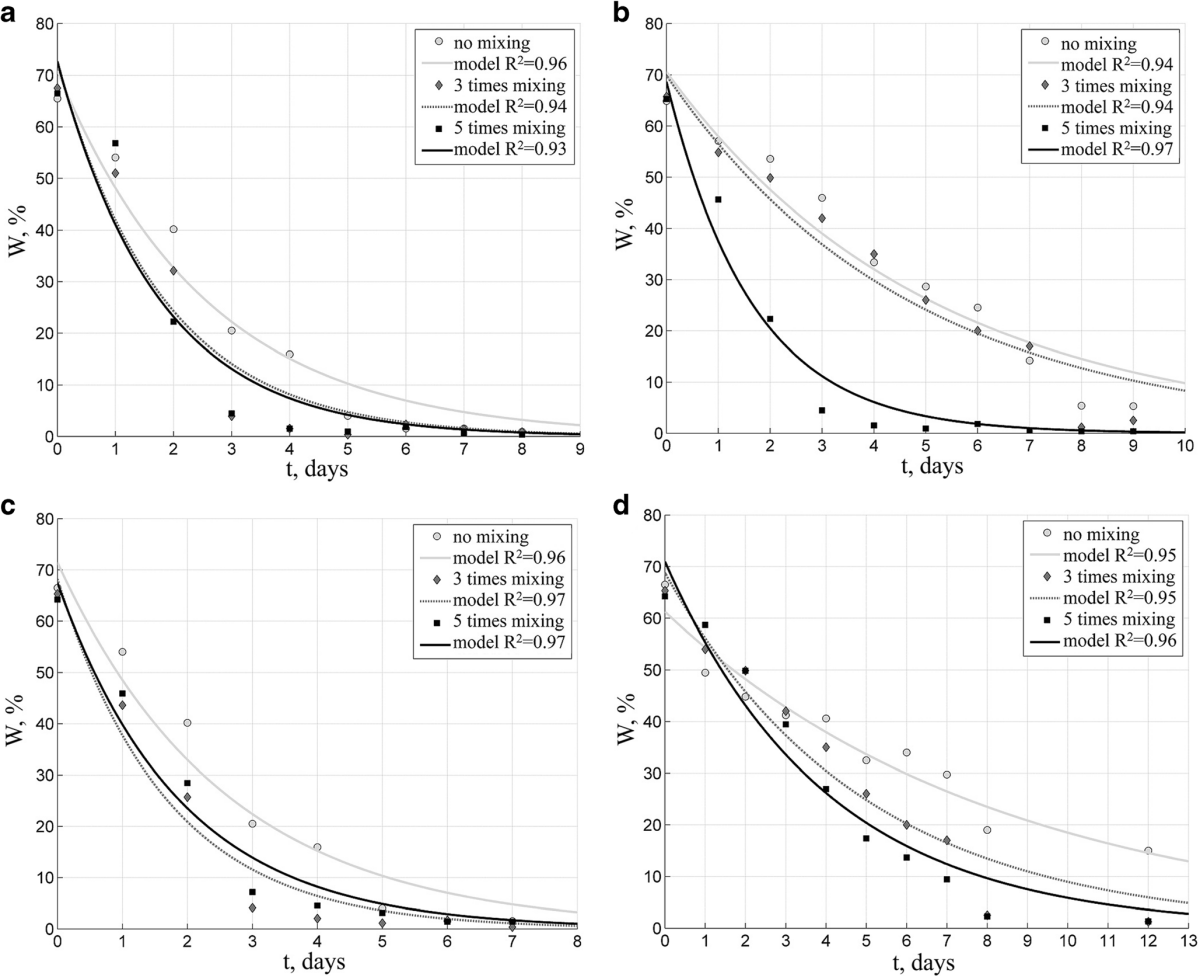
Changes in moisture content of PBT biofuel (W) during solar drying in summer: (**a**) 15-mm granules, 5-cm layer; (**b**) 15-mm granules, 20-cm layer; (**c**) 35-mm granules, 5-cm layer; (**d**) 35-mm granules, 20-cm layer

**Fig. 6:**
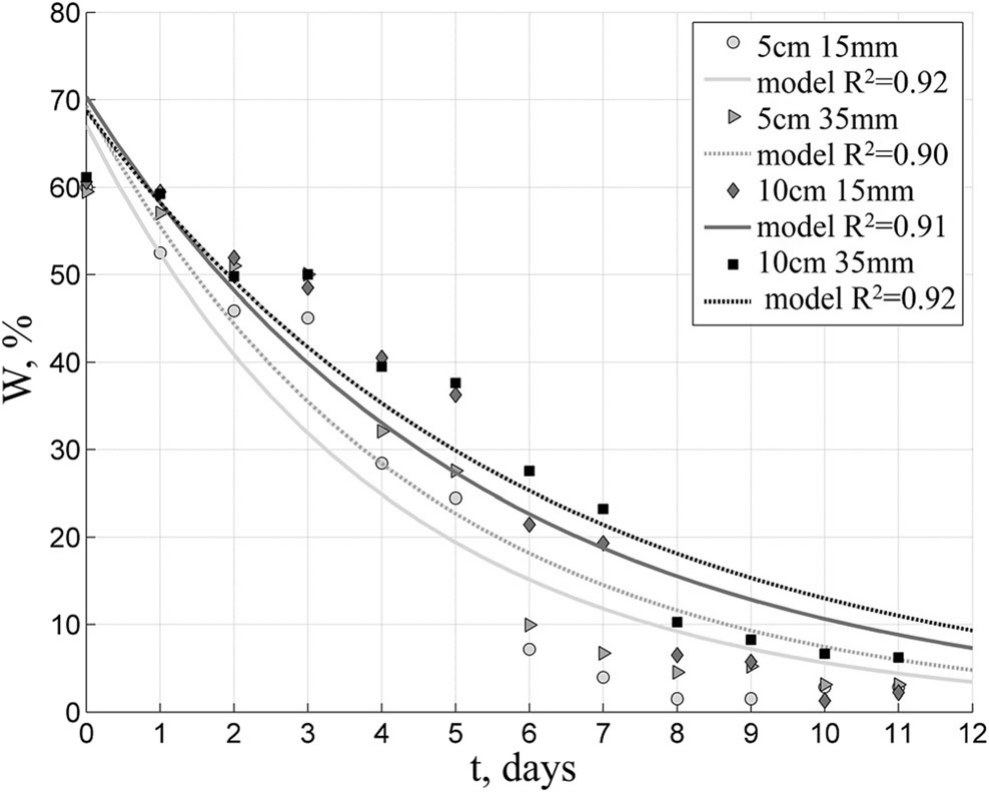
Changes in moisture content of PBT biofuel with 15-mm and 35-mm granules (5- and 10-cm layers) - summer conditions

**Fig. 7:**
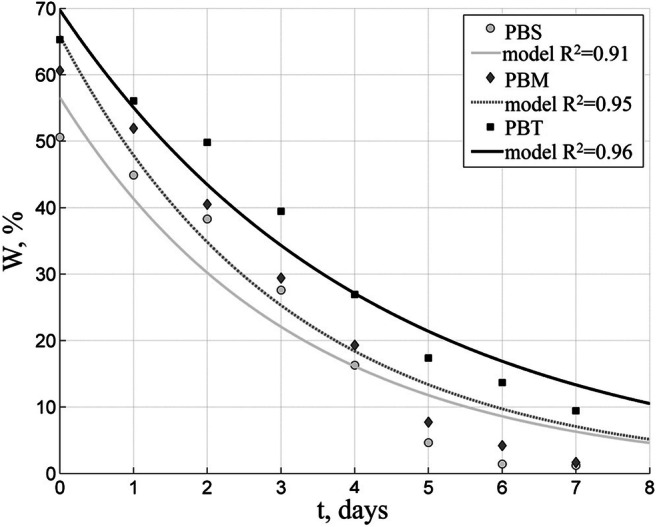
Changes in moisture content of biofuels with 35-mm granules (10-cm layer) - summer conditions

Biofuels were solar-dried at various weather conditions. A series of tests carried out in summer demonstrates the most advantageous conditions for solar drying. In Figs. 3 and 4 are presented weather parameters during the summer tests, and Figs. 5, 6, and 7 show results of solar drying in these conditions.

The mean daily temperature of the drying plant air during that time was 31 °C, the mean temperature between 6 AM and 8 PM stood at 41 °C, and between 8 PM and 6 AM, it dropped to 21 °C. The total mean daily radiation intensity for the period amounted to 241 W/m^2^ and to 340 W/m^2^ during the day. Figure 3 illustrates the temperatures and relative humidity of the air inside the solar-drying plant in summer while the total solar radiation is presented in Fig. 4.

Figure 5 illustrates the process of drying one of the biofuels, i.e., PBT which is characteristic for its highest initial moisture content of 65%, with 15-mm and 35-mm granules dried in layers 5-cm and 20-cm thick during summer.

The drying of PBT biofuel demonstrates that mixing it considerably affects the drying rate. Mixing biofuels with various granulations reduced the drying time by 5 days on the average. When using a thicker layer—20 cm—differences in the drying time of individual biofuels were observed. It took the PBT fuel with 15-mm granules 8 days to reach 10% moisture content; while in the case of PBS and PBM, the time required was 7 days.

A similar effect was observed by Krawczyk (2019) for sewage sludge drying who found that adequate sludge bulk mixing proves more efficient than operation at a reduced sludge layer thickness.

It is due to the fact that mixing facilitates elimination of moisture from inside the biofuel layer being dried, raises the fuel granules from the bottom to the top part of the layer, and thus increases the contact between the newly turned layers and the heated air.

It is well-known that the rate of moisture evaporation depends, among others, on the contact surface of the drying medium and the dried material. The more extended the contact surface, the higher the rate of moisture evaporation may be obtained. The phenomenon was described in the literature *inter alia* by Ruiz et al. (2007), Léonard et al. (2003), and Luboschik (1999).

The drying rate is also improved when the air inside the solar dryer is mixed by the forced draft fans and circulated by the exhaust fans. Segier and Bux (2006) claim that the air mixing is an order of magnitude less effective (per unit of air discharge) than ventilation.

Comparing the size of granules being dried in various layers, on the other hand, demonstrated that their size affects the drying time to a much lesser degree than the thickness of the layer of the fuel being dried.

To illustrate the point, Fig. 6 shows changes in moisture content of PBT biofuel depending on the size of granules being dried in 5-cm and 10-cm thick layers with mixing 5 times/day.

Ten percent of moisture content for PBT biofuel dried in 5-cm thick layers with 5 times/day mixing was obtained after 6 days. The same result for 10-cm thick layers required 9 days of drying.

The performed tests demonstrate that it is advantageous to dry all biofuels in a 10-cm thick layer which is mixed 5 times per day (Fig. 7).

Figure 7 shows that PBS and PBM biofuels required 6 days to reach the 10% moisture content when dried under such conditions, while the PBT fuel needed 8 days.

The mixing systems used in solar drying of sewage sludge use various devices, such as the electric mole which continuously moves haphazardly here and there over the material being dried (**Thermo-system**.Industrie-&Trocknungstechnik GmbH), windrow turners (Veolia 2020), suspended traveling tillers, or self-propelled rotary turners traveling between one and several times across the dryer (Trojanowska 2016). Depending on the mixing technology, they can handle various layer thicknesses of the material being dried.

From an economic point of view, however, drying as much material per area unit as possible seems the best solution; however, to determine the best conditions for the process, one must also consider the process rate, product quality, and prevention of putrefaction processes.

Based on the tests carried out in summer, it was determined that in the case of granulated biofuels, 10-cm thick layers which are mixed 5 times per day give the best results, and tests performed in the fall and spring were designed for exactly such conditions.

**Fig. 8:**
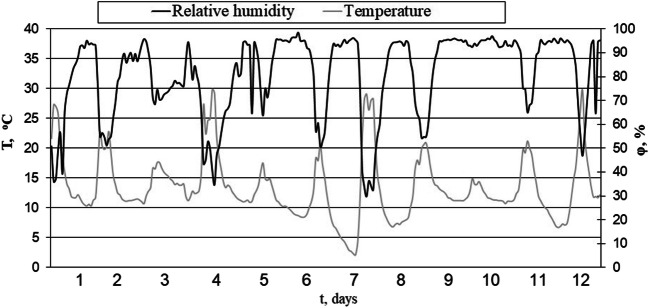
Temperature and relative humidity of the air inside the solar dryer in autumn test

**Fig. 9:**
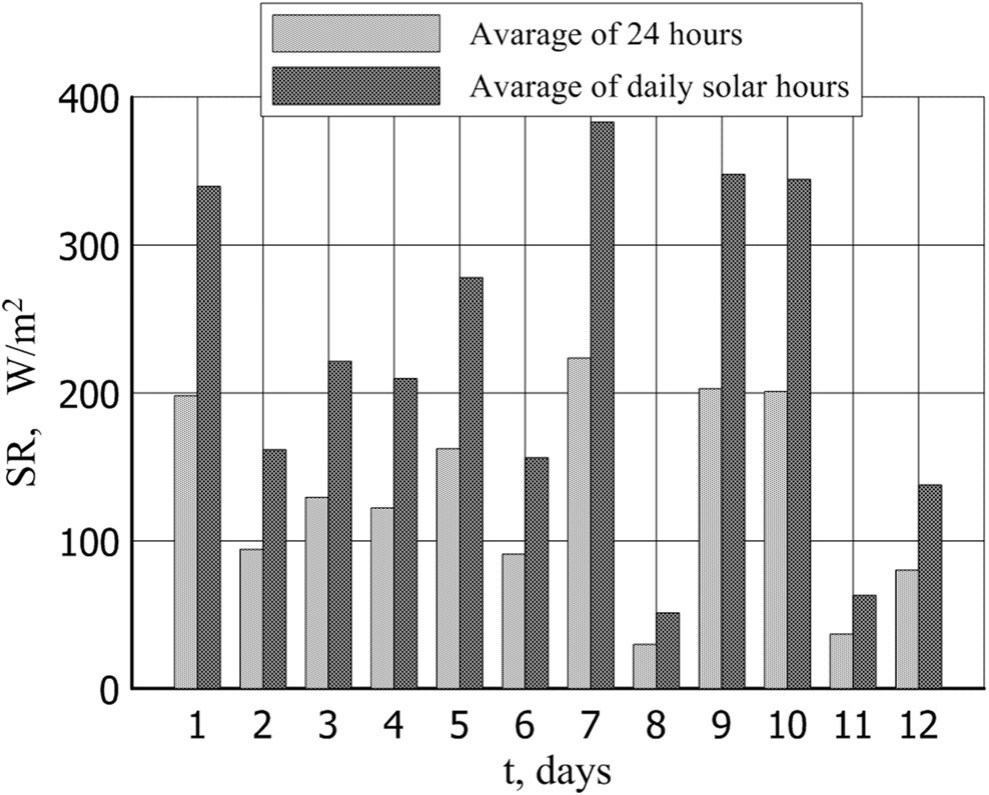
Total solar radiation (SR) in autumn test

**Fig. 10:**
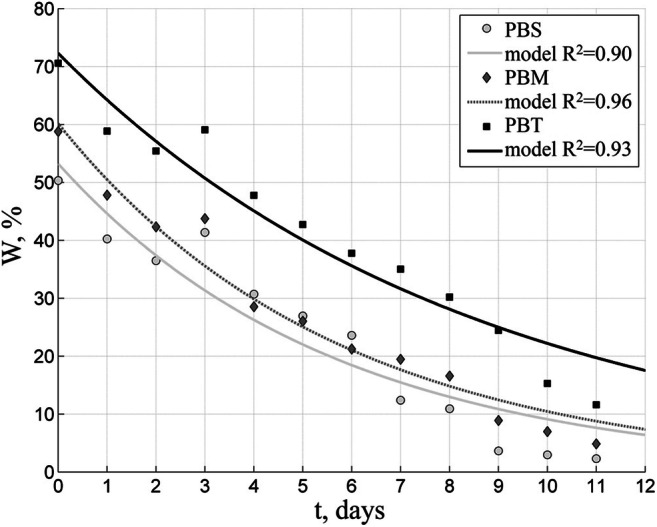
Changes in moisture content of biofuels with 35 mm dried in a 10-cm thick layer in autumn test

**Fig. 11:**
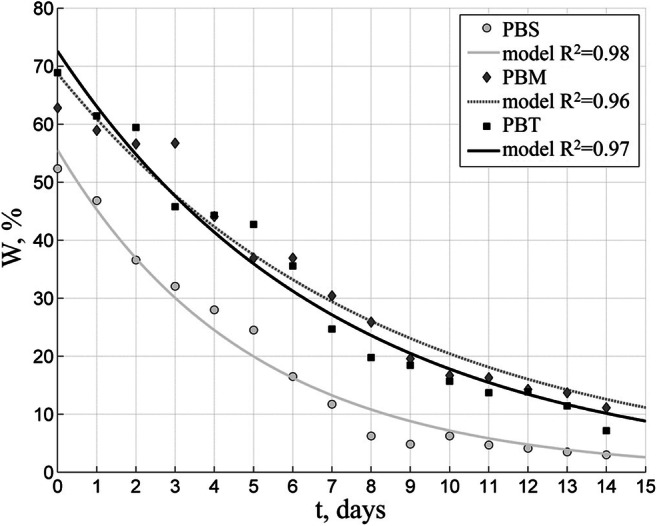
Changes in moisture content of biofuels with 35 mm dried in a 10-cm thick layer in spring test

Figures 8, 9, 10, and 11 illustrate the results of tests carried out in the fall (September/October). Figure 8 shows the temperature and relative humidity of the air at that time, while Fig. 9 illustrates the total radiation intensity. Throughout the entire test period, the average daytime temperature in the drying plant remained at 17 °C, dropping to 10 °C at night. Periods of precipitation observed during that time raised the atmospheric air relative humidity to 80%. The mean daily total radiation intensity for the test period amounted to 135 W/m^2^ and to 231 W/m^2^ during the day.

The curves of drying in autumn (Fig. 10) resemble those obtained in summer, with differences only in the time required to reach the required 10% moisture content; in our case, the time varied from 9 days for the PBS, to 10 days for the PBM, and more than 11 days for the PBT biofuel.

The drying period in spring was characteristic for the lowest temperature of the air (even some cases of temperatures below freezing were observed) and the lowest total radiation intensity. The average air temperature inside the drying plant over that period stood at 12 °C with 69% relative humidity. The mean daily total radiation intensity for the investigated period amounted to 86 W/m^2^ and 171 W/m^2^ during the day. Figure 11 presents the drying results obtained in the spring (March/April).

The spring when the tests were run was unusually chilly, with the lowest air temperatures observed (some of them even below freezing) and the lowest total radiation intensity. Just like during the previous tests, the PBS biofuel demonstrated the fastest moisture reduction, and compared to the fall season, the fuel drying time took longer. The PBS biofuel required 8 days to bring the moisture below 10%, while both the PBM and PBT biofuels took 14 days to do the same. Similar results were obtained when bio-drying sewage sludge: Velis et al. (2009) reported the sludge reached 20% moisture within 7–15 days, while according to Tun and Juchelková (2019), drying municipal solid waste (MSW) in a solar-drying plant may take even up to 38 days at the indoor temperature of 58 °C.

The drying time for sewage sludge alone may also last from a few to more than 30 days. For example, Boguniewicz-Zablocka et al. (2020) report solar drying of municipal sewage sludge containing 88% water and arranged in 5-mm layer under the Polish weather conditions and with outdoor temperature < 20 C takes roughly 4 weeks.

According to Socias (2011), Luboschik (1999), and El-Ariny and Miller (1984) carrying out the research under various climate conditions, it can be concluded that the average daily loss of moisture may ranges from 2 to 17 kg H_2_O/m^2^.

Sobczyk and Sypuła (2011) reports that drying sewage sludge in solar dryers under the Polish weather conditions requires approximately 17–20 kW_t_/Mg of dehydrated sewage sludge, whereas hybrid plants using additional energy require 88–305 kW/Mg for systems featuring a heat pump, and 108–388 kW/Mg for dryers using a heating floor in winter, or roughly 4% of a thermal dryer costs. In the case of biofuels based on sewage sludge, one may assume operating costs and expenses to be comparable to those of typical solar drying of sewage sludge.

The originally designed mixing system together with appropriate operating parameters of the equipment (vertical paddle mixer rpms and turning frequency) does not affect the granule structure negatively. No granule cracking or crumbling has been observed. It may be stated unequivocally that mixing not only accelerates the entire process but also—at the initial drying period when granules still remain plastic—it prevents their sticking and clumping into large agglomerates.

The main purpose of mixing sewage sludge with other components is to increase the energy value and reduce initial moisture of sewage sludge. It is well-known that moisture is a ballast and decreases the fuel energy utility.

Biofuels dried to content of moisture about 10% have lower heating value in the range of 13–19 MJ/kg (Table 2).

**Table 2:** Lower heating value of biofuels from sewage sludge

Parameter	Unit	PBS	PBM	PBT
Lower heating value (LHV)*	MJ/kg	18.83	13.09	13.74

*Calculated: moisture - 10%; hydrogen - 3.91% d.m. (PBS); 4.12% d.m. (PBM); 4.43% d.m. (PBT)

Biosolid fuels have typically a value of LHV like woody and agricultural biomass, which may be in the range of 13–22 MJ/kg (Demirbas et al. 2006). Researches presented by other authors show similar level of calorific value of fuels from sewage sludge compering to tested biofuels. According to Jiang et al. (2016), pellets made from sewage sludge and rice straw have calorific value of 15 MJ/kg and from sewage sludge and unclassified oats above 14 MJ/kg (Junga et al. 2017).

Valorization of physical and energy properties of sewage sludge increases the chances of their use in energy processes.

## Conclusions

Solar drying seems to be an economical solution for pretreatment of highly hydrated wastes for further thermal utilization because requires very low-energy input and allows to obtain the appropriate properties of feedstocks which are favorable for the combustion and transport.

The method for production of various types of biofuels from sewage sludge, such as sewage sludge with coal slime-PBS, sewage sludge with meat and bone meal-PBM, or sewage sludge with sawdust-PBT, makes it possible to choose the type of biofuel to be produced depending on locally available waste.

Based on the tests performed, optimum conditions for the drying process utilizing solar energy were defined for each biofuel, including, especially, the fuel layer thickness plus the method and intensity of the mixing.

It was found that the granule size affected the drying efficiency only to a negligible extent. Another conclusion reached was that the drying process was more effective when the fuel layer was 10 cm thick. It might be expected that assuming the same layer thickness, the water content of the PBS and PBM biofuels would drop below 10% after 8 days both in spring and in fall. During summer, the same result might be achieved for all the biofuels typically after 4 days.

Tests show that winter drying of biofuels is possible only when an additional source of heat is used to support the operation of the solar dryer, such as waste heat of industrial processes or biogas produced in wastewater treatment plants.

In summary, tests support the assertion that an original method for solar drying of biofuels based on sewage sludge and other waste has been developed and may be successfully applied in wastewater treatment plants which process waste into fuels, without incurring any additional costs for the heat supply, as is the case with thermal drying. The method makes it possible to use sewage sludge with a low net calorific value. Mixing it with other appropriately selected components allows to prepare a biofuel suitable for applications for example in cement clinker production.

## Data Availability

Not applicable
